# Non-Invasive Intracranial Pressure Monitoring

**DOI:** 10.3390/jcm12062209

**Published:** 2023-03-13

**Authors:** Sebastian Johannes Müller, Elina Henkes, Matthew J. Gounis, Stephan Felber, Oliver Ganslandt, Hans Henkes

**Affiliations:** 1 Neuroradiologische Klinik, Klinikum Stuttgart, D-70174 Stuttgart, Germany; 2 New England Center for Stroke Research, Department of Radiology, University of Massachusetts, Worcester, MA 01655, USA; 3 Institut für Diagnostische und Interventionelle Radiologie und Neuroradiologie, Stiftungsklinikum Mittelrhein, D-56068 Koblenz, Germany; 4 Neurochirurgische Klinik, Klinikum Stuttgart, D-70174 Stuttgart, Germany; 5 Medizinische Fakultät, Universität Duisburg-Essen, D-47057 Duisburg, Germany

**Keywords:** intracranial pressure, non-invasive ICP measurement, intracranial hypertension, neurointensive care

## Abstract

(1) Background: Intracranial pressure (ICP) monitoring plays a key role in the treatment of patients in intensive care units, as well as during long-term surgeries and interventions. The gold standard is invasive measurement and monitoring via ventricular drainage or a parenchymal probe. In recent decades, numerous methods for non-invasive measurement have been evaluated but none have become established in routine clinical practice. The aim of this study was to reflect on the current state of research and shed light on relevant techniques for future clinical application. (2) Methods: We performed a PubMed search for “non-invasive AND ICP AND (measurement OR monitoring)” and identified 306 results. On the basis of these search results, we conducted an in-depth source analysis to identify additional methods. Studies were analyzed for design, patient type (e.g., infants, adults, and shunt patients), statistical evaluation (correlation, accuracy, and reliability), number of included measurements, and statistical assessment of accuracy and reliability. (3) Results: MRI-ICP and two-depth Doppler showed the most potential (and were the most complex methods). Tympanic membrane temperature, diffuse correlation spectroscopy, natural resonance frequency, and retinal vein approaches were also promising. (4) Conclusions: To date, no convincing evidence supports the use of a particular method for non-invasive intracranial pressure measurement. However, many new approaches are under development.

## 1. Introduction

Measurement of intracranial pressure (ICP) is a pillar for the management of patients in intensive care units. The hypothesis known as the Monro–Kellie doctrine [1,2] states that the intracranial volume remains unchanged because of the limitations imposed by the skull and dura; therefore, the sum of the volumes of the cerebrospinal fluid (CSF), brain, and intracranial blood (venous and arterial) remains constant. Thus, an increase in any of the three components would trigger a decrease in the other components and a pressure difference. Although a natural buffering volume (reserve capacity) is present, when it is depleted, the pressure can rise rapidly. Thus, the possible damage to the brain caused by such a shift can be estimated using the ICP. This pressure has long been considered a static value, but is, in fact, a wave [3] influenced by physiological parameters such as arterial pressure, as well as breathing depth and frequency. The importance of and opportunities for applying continuous ICP monitoring were first described by Pierre Janny in 1950 [4,5] and reiterated 10 years later by Nils Lundberg [6]. Other pioneers of ICP measurement in the 1950s were Goldensohn [7], Ryder [8], and Evans [9,10].

**Figure 1:**
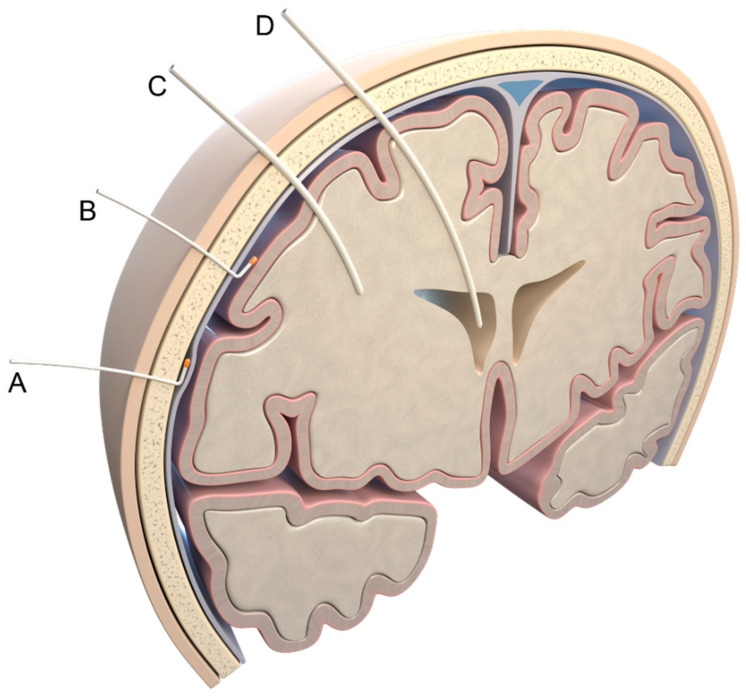
Invasive measurement methods. Possible localizations of pressure probes: (A) epidural; (B) subdural; (C) parenchymal; (D) intraventricular. Illustration created by J. Freimuth.

In principle, the ICP can be measured invasively in three different ways: via lumbar drainage (in a lying position, with high variability) [11], external ventricular drainage [12,13], or a parenchymatous (or epidural/subdural) ICP probe [14,15], as demonstrated in Figure 1. 

All invasive methods carry a risk of infection or intracranial bleeding [16,17,18,19]. Measurements can be taken ipsilaterally or contralaterally to a possible pathology [20]. Another measurement method is continuous measurement after the implantation of a telemetric ICP probe [21,22,23] or a sensor reservoir [24,25]. However, because of the risk of surgery, this method is rarely used for only selected issues, for example, in patients with complex (ventriculo-peritoneal) shunt settings.

Although the ICP is usually given as a static value, in reality, it corresponds to a pulsatile wave, which is influenced by arterial and venous blood pressure, body position, and peritoneal pressure [26], given that direct coupling of the pressure compartments between the brain and the lumbar spine through the spinal canal is often present.

For decades, scientists and physicians have searched for a reliable, non-invasive method for ICP monitoring. To date, although many methods have shown promising results, the necessary measurement precision and reliability according to a Bland and Altman analysis with ground-truth invasive measurements [27] has not yet been achieved in clinical practice [28]. Therefore, invasive measurements are recommended by guidelines [29,30,31] to improve the quality of trauma care [32,33]. Even if zero-shift and calibration errors may occur in solid sensors, the measurement accuracy of ICP waveforms is sufficient [34].

In this review, we describe the current status of non-invasive intracranial pressure measurement and provide an overview of the numerous new methods to identify procedures that may be used in intensive care units in the future.

## 2. Materials and Methods

Our report is a narrative review of methods for non-invasive ICP estimation. This set of heterogeneous measurement concepts can be subdivided according to the device used, the physical properties, or the anatomy of the underlying structures.

**Figure 2:**
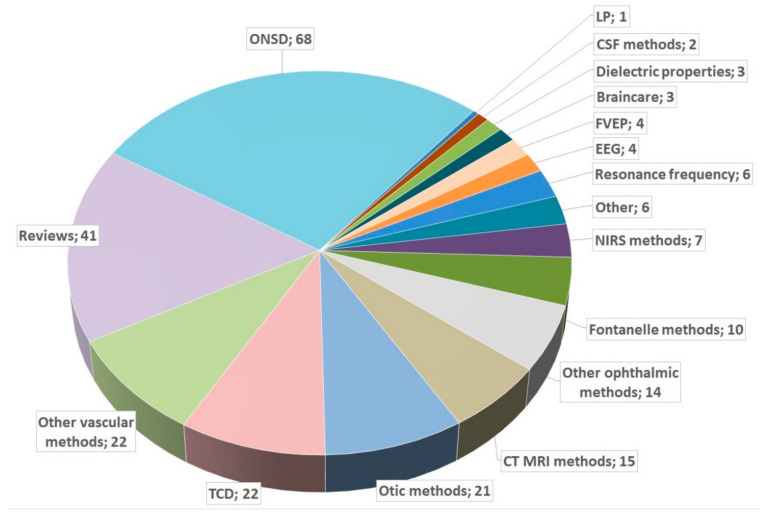
Pie chart of studies in PubMed. ONSD: optic nerve sheath diameter; TCD: transcranial Doppler; LP: lumbar puncture; EEG: electroencephalogram; CSF: cerebrospinal fluid; FVEP: flashed visual-evoked potentials; NIRS: near-infrared spectroscopy; CT: computed tomography; MRI: magnetic resonance imaging.

To provide an overview, we first performed a PubMed search for (“non-invasive” AND “ICP” AND (“measurement” OR “monitoring”)). The 306 results consisted of 68 optic nerve sheath diameter (ONSD) studies, 41 reviews, 22 transcranial Doppler (TCD) studies, 22 other vascular method studies (without TCD), 21 otic method studies, 15 computed tomography (CT)/magnetic resonance imaging (MRI) studies, and 14 other ophthalmic studies (without ONSD). Of the results, 57 studies did not match the content of our search and were excluded. If a study compared two or more methods, it was counted as the rarest method. A pie chart of the included studies is shown in Figure 2.

The main techniques used were ultrasound/Doppler for transcranial or transorbital measurements, CT, MRI, near-infrared spectroscopy (NIRS), tympanic membrane displacement systems [35,36], ear canal temperature and sound probes, dynamic retinal vessel analyzers (DVA, Imedos, Jena, Germany), pillows with mechanical sensors for micromotions [37], ophthalmoscopy, ophthalmic optical coherence tomography, VEP, electroencephalography (EEG), and sensors or headband electrodes to measure impedance changes, acoustic signals, or dielectric properties. 

The measured anatomic structures were vessels, eyes, ears, and the entire skull. The target structures and methods are presented in Table 1.

Six major measurement methods were found: (i) methods based on physiological parameters, (ii) ultrasound/Doppler, (iii) CT/MR imaging-based methods, (iv) di-/electric methods, (v) ophthalmic methods, and (vi) otic methods. Table 2 provides an overview of the number of identified studies and their correlations.

The studies were carried out on phantoms, animal models, and healthy subjects, as well as patients with hydrocephalus and in intensive care units. Patient populations varied widely from infants to adults and from those who were healthy to those with traumatic brain injury (TBI) or hypoxic brain ischemia. 

## 3. Approaches of Non-Invasive Measurements

Several of these methods were tested. However, there remains a strong need for better techniques, particularly for point-of-care use [38].

A much-discussed Lancet article in 1997 postulated a relationship between intraocular pressure and ICP [39]. A correlation between ICP and retinal vein pressure was noted [40], whereas most of the responses were rather dismissive [41,42]. Based on studies of visual impairments in long-term astronauts [43,44], the pressure compartments of the eye and brain are known to be linked, but pressure changes are not transmitted one-to-one between these regions. 

A review of intracranial compliance as a reserve capacity for maintaining ICP stability highlighted the additional potential of this metric in monitoring [45].

In a review from 2012, it was found that non-invasive measurements did not sufficiently and accurately measure the ICP [46]. A recent detailed review advocated for more invasive pressure measurements to facilitate the development of non-invasive procedures [47] because a novel non-invasive measurement method should not be calibrated using an older, non-invasive, unreliable measurement method. 

Another review found that no non-invasive methods were suitable on their own and recommended the use of a flow chart to evaluate the various non-invasive methods to avoid the placement of cranial pressure probes [48]. Many other reviews have also recommended continued invasive pressure measurements and the further development of non-invasive methods [3,49,50,51,52]. One review noted the high accuracy of ONSD in estimating the ICP [53].

In a recent study, the neurologic pupillary index was found to have the highest prognostic value for short-term unfavorable outcomes in patients with TBI but recommended a combination of several methods overall [54].

In a meta-analysis of critically ill patients from 2019 [55], the pooled sensitivity and specificity for increased ICPs was 85.9% for pupillary dilation, and the pooled areas under the receiver operating characteristic (ROC) curve for increased ICPs were 0.94 (0.91 to 0.96) for ONSD sonography and 0.55 to 0.72 for the TCD pulsatility index.

A study of hypoxic brain ischemia indicated a moderate correlation with the ICP [56]. The study found that ONSD (r = 0.53), jugular vein pressure (r = 0.38), and TCD flow velocity (r = 0.30) all had some correlation with ICP. However, these measures had a high ability to detect an elevated ICP with areas under the ROC curve values of 0.96 for ONSD, 0.91 for TCD, and 0.75 for jugular vein pressure.

It must be noted that most studies only report correlations. Although the Pearson’s correlation coefficient is a useful statistical method, it provides only a rough estimate and may not represent a reliable determination of the accuracy and reliability of a measurement method. Therefore, more stringent statistical methods (e.g., Bland–Altman plot) should be used instead.

### 3.1. Methods Based on Physiological Parameters and Ultrasound/Doppler

The importance of monitoring multiple values in the complex treatment of patients in intensive care has been confirmed by the measurement of the cerebral perfusion pressure (CPP), which can be calculated according to the difference in the mean systemic arterial blood pressure and the ICP [57]. In the supine position, an optimal CPP between 50 and 70 mmHg and an ICP lower than 20 mmHg are desirable [58]. The normal CSF opening pressure measured by lumbar puncture in the lying position is 15 mmHg or lower This value depends on the body mass index [59] and agrees with the ICP [60]. In the sitting and standing positions, the intracranial pressure is lower than the lumbar CSF pressure. 

A refractory elevated ICP is associated with significantly elevated odds ratios (OR) for death (20–40 mmHg: OR 3.5; >40 mmHg: OR 6.9) [61].

Correlations between the ICP and single physiological parameters have been evaluated but have not shown promising results. For example, a waveform analysis of the radial arterial blood pressure has indicated a good correlation with ICP waves in approximately one-third of patients. Because of the high variance, this approach is not reliable in clinical practice [62].

#### 3.1.1. Transcranial Doppler

The concept of an increasing ICP correlating with changes in transcranial propagation of ultrasonic impulses was described in the late 1980s [63,64] and in 1992 [65,66]. In multiple studies (1994–2001), waveform analysis of TCD blood flow velocity in the middle cerebral arteries revealed a high potential for the monitoring of the CPP [67,68,69,70]. 

A review found a broad range of prediction accuracies (area under the curve values) from 0.62 to 0.92 in the detection of elevated ICPs (≥20 mmHg) [71]. The main methods are based on the correlation between the ICP and TCD-derived pulsatility index (a method with relatively low reliability) [72,73] or the direct estimation of the CPP or multiparametric/model-based ICP calculations. Such algorithms use frequency and time domain analysis based on the cross-correlation of the non-invasive blood flow of the middle cerebral artery with blood pressure measurements, as well as the phase shift [74,75]. Examples of calculation models were described by Ursino [76,77,78] and Kashif [79]. A model based on resistance circuits and hemo- and hydro-dynamics was described by Lee [80].

An advantage of TCD measurements is that they are easy to obtain and perform, but only an optimal ultrasound window through the cranial bones can be found rapidly. The results also depend on the ultrasound operator. The quality of ultrasound devices has substantially improved in recent decades [81].

A retrospective evaluation found a moderate correlation (r = 0.51) between the ICP and non-invasive ICP, calculated using the middle cerebral artery blood flow velocity and arterial blood pressure [82]. An algorithm based on an unscented Kalman filter (using arterial blood pressure, and cerebral blood flow and velocity measured by TCD) demonstrated an improved correlation (r = 0.75) [83].

A fuzzy logic algorithm has also been applied to TCD and arterial blood pressure [84], and support vector machines [85] have also been used.

In a recent prospective study, the diastolic flow velocity showed a good correlation (r = 0.72) with the ICP [86]. 

A study of patients with suspected idiopathic intracranial hypertension demonstrated excellent results for detecting increased ICPs, with a sensitivity and specificity of 0.92 for a cutoff of approximately 15 mmHg (20 cmH_2_O) [87]. Currently, several commercial tools are available for non-invasive calculations of the ICP from the TCD, flow velocity, and arterial blood pressure signals. 

(Ophthalmic Doppler methods are presented in Section 3.4).

#### 3.1.2. Near-Infrared Spectroscopy

In 1985, early experiments on cats suggested that near-infrared spectrophotometry could provide additional data on the ICP and brain metabolic activity [88]. A pilot study in 1997 [89] found significantly lower NIRS values in patients with TBI and an ICP > 25 mmHg compared to those with an ICP < 25 mmHg. 

The hypothesis that an exhausted pressure compensatory reserve of the brain is indicated by increased slow-wave activity in the ICP was evaluated in a study showing correlations between these slow waves of the ICP and NIRS values during the controlled elevation of ICP via infusion tests [90] and observations in TBI [91]. A moderate correlation between cerebrovascular pressure reactivity and NIRS has been shown (r = 0.56 across patients, r = 0.49 averaged individual recording) [92].

The limitations of this method appear to be primarily due to the need for a sufficiently slow wave. Diedler et al. found correlations of r = 0.80 (good) and r = 0.07 (no correlation) in an analysis of subgroups with normalized vs. non-normalized slow-wave activity [93].

Even better results with an optimized wave analysis and an increased correlation (r^2^ = 0.86 for NIRS and r^2^ = 0.91 for diffuse optics) were shown in a recent study of primates under controlled conditions [94].

In a recent pediatric study, a correlation between an elevated ICP and ONSD but no correlation between ONSD and NIRS was demonstrated [95].

An NIRS-associated technique, so-called diffuse correlation spectroscopy, is a variant of the dynamic light-scattering method [96]. A pilot study using this technique revealed an excellent correlation (Pearson’s correlation coefficient > 0.9) and concordance (Lin’s concordance correlation coefficient > 0.9) [97]. Overall, promising results were found, including through a Bland–Altman analysis, but the number of patients was small.

Overall, these methods do not yet appear to be mature and further validation studies are required.

#### 3.1.3. Combined Methods

A review of multimodal monitoring reported a pooled correlation of r = 0.72 between the TCD and ICP and recommended the similar use of the TCD for ICP control and NIRS for brain oxygenation monitoring in patients with TBI [98].

In a recent prospective study, ONSD measurements, as well as the diastolic flow velocity, demonstrated a good correlation (r = 0.76 and r = 0.72, respectively), and a combination of both methods showed even better performance (r = 0.8) [86]. 

A review of minimally invasive multimodal monitoring was conducted [99].

A recent study [100] observed patients during spinal surgery and found a correlation between the ICP and the patient’s position (prone versus supine), as well as the use of positive end-expiratory pressure, ONSD, and TCD values. The study found that ONSD had the strongest single correlation with the ICP (r = 0.75), and successful implementation of artificial neural networks allowed for multiparameter analyses.

Most methods can be improved by regular calibration [101] based on constant shifts or by model reconstruction [102]. A study from Spain analyzing the complexity of ICP signals suggested that periods of intracranial hypertension can be detected from non-invasive parameters, e.g., pulse oximetry [103].

A multiparameter fusion model using vital and hemodynamic parameters, TCD, and FVEP found an excellent correlation with an invasive ICP (r = 0.931) [104].

Software called ICM+ was developed by Cambridge University to handle the complexity of intensive care monitoring, including the estimation of CSF dynamics and ICP [105].

#### 3.1.4. Fontanelle in Newborns and Infants

A special case for measuring the ICP exists in newborns, whose fontanelles provide an additional possibility for measuring pressure. In 1980, the first non-invasive measurements of the ICP in infants via the anterior fontanelle were performed [106,107,108]. During the 1980s, this method was developed in different parts of the world, for example, in Japan [109,110,111] and later in England [112]. Other measuring devices developed during that time such as the Rotterdam transducer were also used for these measurements [113,114]. The conclusion of most studies was that good fixation of the sensors on the scalp is necessary for reliable measurement. Currently, standard ICP probes can be fixed epicutaneously on the skin over the frontal fontanelle and can reliably measure ICP values [115,116]. In 2021, a new bandage-like sensor was introduced, with an excellent correlation between the measured values and the real ICP (r^2^ > 0.9990) [117].

#### 3.1.5. CO_2_

The hypothesis that CO₂ levels influence the ICP and can be used for non-invasive measurements was tested through combined exposure to CO₂ and head-down tilt. The results indicated only small effects on an increasing ICP [118].

#### 3.1.6. Micromotions of the Skull/Mechanical Extensometer

The concept of the waves of the ICP reaching the skull and causing expansion and retraction, which can be measured as a pulse, was first described by Ueno [119]. A novel device (Braincare™ Inc., Sao Paulo, Brazil) based on this concept was preliminarily evaluated in patients with HIV-associated cryptococcal meningitis [120], TBI [121], and idiopathic intracranial hypertension [122]. In experimental studies in dogs, similar results were achieved [123]. In a recent study [124], this mechanical extensometer achieved excellent results in estimating the pulse amplitude index. 

Another similar device (Charles University in Prague and Military University Hospital, Prague, Czech Republic), a specially developed pillow for detecting micromotions/head tremors, also achieved good results (r = 0.74) in initial clinical testing [37]. 

#### 3.1.7. Other Approaches: Eigenfrequency, Microwaves, and Ultrasound

The (acoustic/vibration) natural resonance frequency (NRF) spectrum and its modal damping characteristics were successfully used to estimate the ICP in an experimental study in rabbits [125]. An excellent correlation was observed between the best NRF and the inserted CSF volume (r^2^ = 0.96) as a surrogate for the ICP. An interesting study from Japan [126] suggested that the NRF depends only on the ICP and that the relationship between the ICP and the NRF in the brain can be calculated using a quadratic function (ICP = 0.0329 × NRF^2^ + 0.0842 × NRF), with an excellent correlation (R^2^ = 0.9952). Therefore, the individual NRF depends only on the ICP value. 

Innovative approaches based on microwaves [127] and high-frequency ultrasound [128] have been introduced and have shown excellent preclinical test results. However, further clinical results are needed to assess their effectiveness in clinical settings.

In a prospective study, cerebrovascular autoregulation was monitored invasively and non-invasively using arterial blood pressure and intracranial blood volume slow waves (reflecting vasodilation and constriction of small vessels), detected by a pair of ultrasonic transducers on either side of the patient’s head [129].

### 3.2. Computed Tomography (CT) and MR (Magnet Resonance) Imaging-Based Methods

#### 3.2.1. CT

CT imaging plays a minor role in non-invasive ICP studies. Although bleeding and a midline shift can be detected, which primarily reflect an increased ICP, the absence of these two findings does not indicate the absence of an increased ICP. In a meta-analysis [55], the following mean sensitivities and specificities for the observed ICPs were found: 85.9% and 61.0% for compression of basal cisterns, 80.9% and 42.7% for any midline shifts; and 20.7% and 89.2% for substantial midline shifts (at least 10 mm). 

#### 3.2.2. MR Methods

MR technologies have been used to estimate the CSF velocity in the aqueduct and foramen magnum so that craniospinal CSF pressure gradients can be calculated. An additional parallel measurement of cranial fluid flows during a cardiac cycle (CSF flow, arterial inflow, and venous outflow) enables the estimation of cranial compliance and elasticity. 

**Figure 3:**
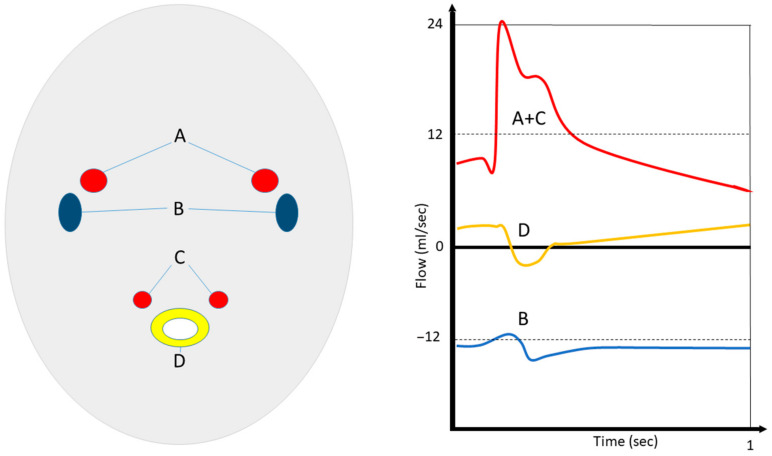
Concept of MR-ICP. Simultaneous (in and out) flow measurements of internal carotid (A) and vertebral (C) arteries, as well as venous flow in the jugular veins (B) and cerebrospinal fluids at the C2 level (D), enable the estimation of cerebral compliance, pressure gradients, and ICP. (Schematic overview made by S.J. Müller).

This method was introduced in 2000 and was called MR-ICP [130]. It indicated substantial agreement (r = 0.965) between the estimated non-invasive ICP and the invasively (intra-ventricular) measured ICP. In another study, these results were confirmed (r = 0.95) [131]. In 2005, this method (also called cine MRI) was successfully used to determine intracranial compliance in patients with normal-pressure hydrocephalus, according to this concept [132]. Combined MR ICP and MR flow (phase contrast for internal carotid arteries, vertebral arteries, internal jugular veins, and CSF flow; T2*-weighted, sagittal gradient echo, axial time of flight) techniques in patients with intracranial hypertension have demonstrated promising results [133]. An illustration of this concept is shown in Figure 3.

The main idea, which is based on Bernoulli’s principle and a Fourier transform, was demonstrated by Muehlmann et al. [134], who showed the feasibility of this method in infants with a ventriculo-peritoneal shunt. 

In a pilot study, the pulse pressure gradient of the spinal canal at level C2 assessed using phase-contrast MRI (using a simplified Navier–Stokes equation) demonstrated no correlation with the pulsatile ICP [135].

New real-time MRI technologies [136] suggest that breathing also plays an important role in the correct estimation of lumbar and intracranial pressure. Additionally, the novel mapping [137,138] and FLASHlight [139] methods may also provide new approaches for non-invasive ICP measurements. 

### 3.3. Electrophysiological Properties

#### 3.3.1. Dielectric/Electric Impedance Methods

In 1932, Atzler and Lehmann [140] introduced a technology called dielectrography and described its use in measuring heart activity. The basic concept is that the movement of blood and the resultant capacity changes between two capacitor plates can be measured.

The intracranial electrical impedance method (or “rheoencephalography”, “impedance cephalography” or “impedance plethysmography”) measures blood flow through an alternating current and was introduced for neurologic diseases in the early 1960s by Jenkner [141]. Initial studies indicated no meaningful clinical applicability, as well as excessive inaccuracies and confounding variables [142]. 

In the early 1990s, a model device (headband) for measuring dielectric properties was described by Russegger and Ennemoser [143] and showed excellent results (r = 0.983). In 2003, the dielectric properties of the human brain were analyzed postmortem [144]. In 2005, a study in rats demonstrated the potential of this method at the lower limit of cerebral blood flow autoregulation [145]. The idea was revived in the recent introduction of a similar skin patch approach [146]. A simplification of the model by removing high-frequency poles has been suggested [147].

A novel, high-precision electrical impedance tomography method introduced in 2018 [148,149] uses electrical conductivity, permittivity, and impedance to form a three-dimensional image of the brain.

A study from Scotland examined the relationship between electrical impedance and ICP and concluded that electrical impedance is not suitable for reliably estimating the ICP [150]. An article from 2012 demonstrated the basic suitability of the method but also its high susceptibility to disruptive factors such as breathing [151].

When electromagnetic waves pass through the head and brain, they are influenced by the dielectric properties of the CSF, brain, and blood. On the basis of this idea, a pilot study in rabbits revealed the potential of broadband antennas to detect intracranial hemorrhage, with an accuracy of 77% [152]. Another study in a rabbit model introduced an electromagnetic coupling phase-sensing technology with an AUC of 0.88 for detecting an increased ICP [153,154].

#### 3.3.2. Electroencephalography (EEG)

In retrospective feasibility studies, a strong causal relationship between EEG and the ICP was identified [155,156] and may be used for devices in the future.

In a swine model, the best estimation of single-channel EEG signals with ICP was found using a support vector machine [157], with a correlation of 0.773.

### 3.4. Ophthalmic Methods

Reviews of methods of non-invasive measurements of ICP via ocular measurements highlighted the limitations and potential of specific applications [158,159] but emphasized that these methods cannot fully replace invasive measurement techniques.

**Figure 4:**
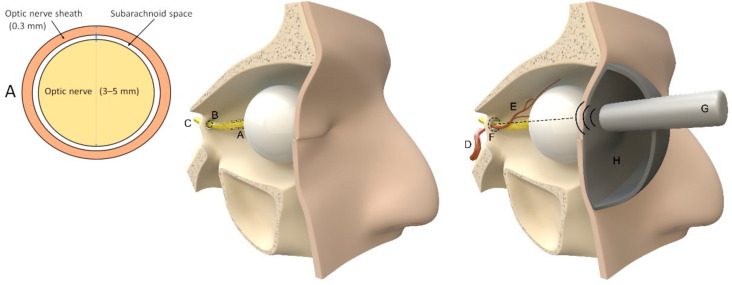
Ophthalmic methods. Left: schema of a coronary slice of the optic nerve 3 mm behind the globe (A); middle: illustration of intracranial (C) and extracranial (A) course of the optic nerve through the annulus of Zinn and the optic canal (B). Right: abstract illustration of ONSD and two-depth Doppler (G) measurements of intraorbital (E) and intracranial (F) ophthalmic artery branching from the internal carotid artery (D), following the descriptions of Raugaskaus with a pressure pad (H) on the eye. Illustrations were made by J. Freimuth.

Another review [160] reported the following sensitivity and specificity values: ultrasound (37–100%, 58–100%); MRI-ONSD (90%, 92%); MRI-ICP (100%, 100%); two-depth-TCD (68–73%, 77–84%); and optical coherence tomography (98%, 62%). The concept of ONSD measurements and two-depth Doppler is illustrated in Figure 4.

#### 3.4.1. Ophthalmic Artery and Ophthalmic Arterioles

Ragauskas et al. [161,162,163,164] introduced a two-depth Doppler approach with excellent accuracy and reliability in initial studies (including a Bland–Altman-Plot). The approach uses a preocular, extracranial pressure chamber, as well as a two-depth transorbital Doppler aimed at the intracranial and extracranial segment of the ophthalmic artery. No calibration through invasive measurements is required. In a prospective study, the approach performed better than ONSD measurements in detecting increased ICPs [165]. In recent studies, good (r = 0.74) [166] and excellent (r = 0.94–0.99) correlations were demonstrated [164,167]. An updated ICP measurement method for patients with glaucoma used the two-depth Doppler method [168]. 

The frequency of the oculocardiac reflex as a disturbance variable in measurements [169] in ophthalmic examinations is low and can be considered negligible.

Another approach in 2020 using fundus photography to calculate the ratio of the arteriole and venule diameter was described [170], revealing a 94% sensitivity and 50% specificity for detecting elevated ICPs. In patients with an ICP > 15 mmHg, a significantly negative correlation between the ICP and arteriole and venule diameter ratio was found.

#### 3.4.2. Retinal Vein Pulsation

Ophthalmodynamometry was introduced by Bailliart in 1917 [171]. Elliott and Baurman analyzed the phenomenon of retinal vein collapse in the 1920s and proposed a connection between retinal venous pulses and the ICP. A strong correlation between the intraocular pressure at which the veins collapse and the ICP was revealed 80 years later [40,172]. The concept was further developed and combined with photoplethysmography [173]. Modified photoplethysmography was also tested as a stand-alone method for predicting the ICP [174].

Venous ophthalmodynamometry was successfully tested for ICP estimation on Mount Everest (r = 0.85) [175] and in idiopathic intracranial hypertension [176].

A novel method for objective detection of venous vessel pulsation through high-resolution video recordings was introduced in 2015. This method may help to improve current measurement methods [177]. An automatized approach achieved an accuracy of 77% in the estimation of the ICP [178]. 

A model for a better mathematical understanding of these pulsations was described by Morgan et al. [179]. 

A model based on zero retinal vein pulsation measured by photoplethysmography showed an excellent correlation with an invasive ICP (r = 0.91) [180].

The mechanism underlying the effects of an increased ICP on retinal veins consists of increasing the cerebral venous pressure, elevating the CSF pressure in the optic nerve sheath, and developing papilledema. Therefore, changes in retinal veins can be used to indicate an increased ICP [181].

Multi-layer perceptron neural networks with input vectors of retinal vein pulsations and intraorbital pressure demonstrated excellent accuracy and reliability [182] for the non-invasive estimation of the ICP, but only in a limited group of 15 probands and only in comparison to another non-invasive device (ICM+).

Larger prospective studies on these promising mechanisms are needed. 

#### 3.4.3. Ultrasound of ONSD

The mean diameter of the optic nerve in healthy patients is 3 mm and the optic nerve is surrounded by a small subarachnoid space (0.1–0.2 mm thickness) and a leptomeningeal sheath (0.3 mm thickness) [183,184]. A direct connection between the subarachnoid space of the optic nerve and the chiasmatic cistern exists, thus allowing for the communication of the CSF between these compartments [184]. In a large meta-analysis [185,186], the threshold for the optic nerve sheath diameter was found to be 5.0 mm. Higher values may indicate an increased ICP and additional tests should be performed. A study in healthy volunteers from Canada [187] revealed a median ONSD of 3.68 mm (95% confidence interval, 2.85–4.40). Contradicting earlier studies, a large study in healthy Chinese people found a median ONSD of 5.1 mm, with a 95% percentile of 5.9 mm [188]. 

ONSD is position-dependent [189]. In most studies, measurements were performed in a standardized manner, with the patient in a supine position, the probe directed orthogonally to the optic nerve axis, in two planes for each eye, and at a distance of 3 mm behind the globe [190]. Another study from Germany [191] reported a mean ONSD of 5.4, with an interrater correlation of 0.81. Measurements are highly dependent on the examiner, even if the given inter-rater reliability in studies is reported to be excellent, e.g., with an intraclass correlation coefficient of 0.82 among three ultrasound-trained emergency physicians [192]. In a single-operator prospective study, an ONSD ≥ 5.2 mm had a sensitivity of 83.3% and a specificity of 100% in detecting elevated ICPs [193]. Nevertheless, the serial recording of ONSD in head injuries appears particularly valuable [194]. 

In 1987, increased ONSD was observed in patients with intracranial hypertension [195]. In 2002, ONSD was successfully used to detect shunt dysfunctions in children with hydrocephalus [196]. 

In a meta-analysis, ONSD was found to have an ROC AUC of 0.932 in detecting increased ICPs (>20 mmHg), with a sensitivity of 88–95% and a specificity of 74–96% [197].

An interesting study on asymmetric ONSD suggested that this method lacks significant diagnostic value [198].

A study of children indicated a sensitivity of 93% and a specificity of 74% in detecting increased ICPs with a threshold ONSD of 5.5 mm (subgroup threshold for infants <1 year 5.16 mm, and >1 year 5.75 mm), with excellent inter-rater reliability [199,200]. The control group in another study of children with TBI [201] showed a median ONSD of only 4.5 mm [4.1–4.8]. In another study, an ONSD > 4.5 mm was rated as abnormal [202]. A prospective blind study of patients with either external ventricular drains or a parenchymal ICP probe reported an optimal ONSD of 4.8 mm, with a sensitivity of 96% and a specificity of 94% [203]. A study from China found a cutoff of 4.1 mm for an increased ICP, with a sensitivity of 95% and a specificity of 92% [204]. In a study comparing only color Doppler and B-mode, the first measurement method indicated significantly lower diameters [205]. This finding, along with the use of different ultrasound devices, may explain the differences between studies.

In adults, ONSD demonstrated a good correlation with invasive ICP measurements of r = 0.71 [206], r = 0.53 [207], r = 0.60 [208], r = 0.82 [209], and r = 0.61, and excellent diagnostic value in detecting an ICP > 20 mmHg, with a mean sensitivity and specificity of 85% [210]. 

In one review, the typical cutoff values were summarized as 5.0 mm in adults (>15 years of age), 4.5 mm in children 1–15 years of age, and 4.0 mm in infants <1 year of age [211].

Case reports have described the successful clinical application of ONSD-only monitoring under various conditions, including anticoagulation [212], intraoperative monitoring [213], and point-of-care testing [214].

Under general anesthesia, the strong effects of carbon dioxide levels were observed in children [215] and adults [216]. A decreasing CO^2^ level was correlated with a decrease in ONSD. Another study of awake patients with ICP reported a threshold of 5.05 mm, with a sensitivity of 92% and a specificity of 90% [217].

An experimental study revealing that ONSD decreases with lumbar puncture also demonstrated the ability to collect real-time ONSD measurements [218]. Similar results were shown by studies before and after ventriculo-peritoneal shunt surgery [219] and during surgery [220].

In a swine model with intracranial balloons, excellent correlations between ONSD and balloon volume, as well as invasively measured ICP, were found [221].

A study of patients with idiopathic intracranial hypertension indicated a correlation between ONSD and body mass index [222].

In a pilot study, the measurement of (only) the subarachnoid space of the optic nerve achieved better results than ONSD [223].

Pocket-sized ultrasound devices have also been evaluated [224,225] and have shown moderate results for accuracy in addition to device and manufacturer-dependent measurement errors. ONSD measurements were also considered in space flight [226]. A study observing the shear-wave elastography of the optic nerve found similar correlations of ONSD and Young’s modulus (E) with the ICP [227].

In the future, the standardized automated assessment of ONSD [228] is expected to enable serial monitoring, as well as cross-comparisons between populations.

#### 3.4.4. CT and MRI of ONSD

Owing to the transport risk in patients in intensive care, monitoring using MRI or CT is scientifically interesting but clinically less important. In MRI, the optic nerve appears isointense to cerebral white matter in T1- and T2-weighted images and is surrounded by the optic nerve sheath containing the pia, CSF, arachnoid, and dura [229]. In general, T2-weighted fast spin-echo sequences with fat suppression performed best [230], followed by contrast-enhanced T1-weighted fast spin-echo fat-suppression images [231]. Even if a coronal image appears optimal for assessing the nerve sheath, studies typically combine coronal and axial images.

Beyond an optic nerve sheath with increased diameter, several other properties of revealed ICP have been found: (i) a flattening of the posterior sclera, (ii) papilla protrusion, and (iii) kinking or tortuosity of the optic nerve [231].

In principle, MRI is superior to CT in detecting optic nerve sheath diameter owing to the better representation of soft tissue and water. However, a substantial agreement (r = 0.959) between measurements of the optic nerve sheath diameter using CT and MRI was demonstrated in patients with craniosynostosis [232].

High-resolution MRI indicated an additional correlation with body mass index and demonstrated a sensitivity of 70% and a specificity of 72% in the prediction of increased ICPs using a model based on MRI and the body mass index (BMI) [233].

#### 3.4.5. Ophthalmoscopy

An ophthalmoscopic study revealed excellent sensitivity (100%) in the detection of elevated ICPs but poor specificity of only 32% [234]. Several observations may be indicative of an increasing ICP, e.g., blurred vision, hemorrhage, papilla elevation, ocular venous swelling, optic nerve dilatation, and retinal venous tortuosity [234]. This procedure can, therefore, only be used as a method to rule out other conditions.

#### 3.4.6. Optical Coherence Tomography

Optical coherence tomography can be used to detect a thickening of the retinal nerve fiber layer and, therefore, can be used as a quantitative tool to detect early papilledemas [235] and, consequently, increased ICP. In a pilot study, ICP changes after lumbar puncture were detected in five patients using this method [236].

#### 3.4.7. Visual-Evoked Potentials

Visual-evoked potential latency can be delayed in patients with idiopathic intracranial hypertension and can detect optic nerve damage or increased ICP [237]. Hence, a linear correlation with a large error has been assumed, and use in ICP monitoring in combination with Doppler techniques is considered possible [238]. However, FVEP alone is not reliable [239].

#### 3.4.8. Pupils

The main clinical application of portable pupillometry, which was introduced in 1989, remains the assessment of brain stem function [240].

A neurological pupil index measuring pupillary reactivity was introduced in 2012 [241] but has only demonstrated a trend toward an inverse correlation of increased ICP with a decrease in pupil reactivity. An association between unfavorable 6-month outcomes and diminished pupil reaction was reported [242]. Nevertheless, the method overall does not appear to be suitable for reliable monitoring.

### 3.5. Otic Methods

**Figure 5:**
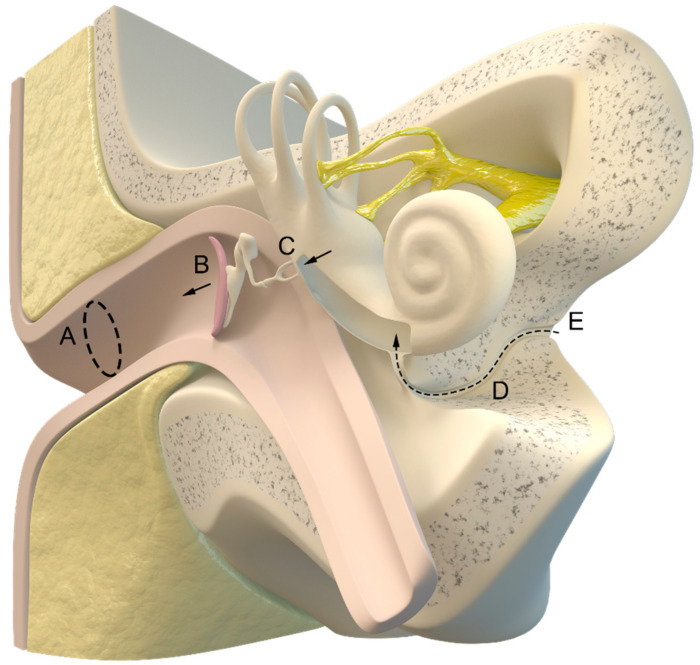
Illustration of the inner and outer ear with the cochlear aqueduct (D) connecting the intracranial cerebrospinal fluid (E) and cochlea. (A) ear canal, (B) tympanic membrane, (C) auditory ossicles. Illustration made by J. Freimuth.

The idea of using intracochlear fluid pressure as a possible monitoring device was described in 1987 [243], and the first pilot study for the so-called “tympanic membrane displacement technique” that was performed 2 years later indicated good results in young patients with accurate stapedial reflex and a patent cochlear aqueduct [35,36]. An anatomic illustration of the involved structures is shown in Figure 5.

#### 3.5.1. Tympanic Membrane and Cochlear Microphonic Potential

Tympanic membrane displacement initially showed excellent results in patients with ventriculo-peritoneal shunts [244], as well as in children (sensitivity of 93% and specificity of 100%), in predicting ICP changes [245]. In later studies, this test demonstrated only limited success [246]. The idea that the cardiovascular pulse can interfere with the measurements and cause high measurement variabilities was analyzed [247]. Reference intervals [248], as well as refining techniques, [249] were reported. 

Other pilot studies analyzed otoacoustic emissions [250,251,252,253,254,255], tympanic membrane pulsation waveforms [256], and cochlear microphonic potential [257,258], and demonstrated moderate correlations with the ICP. Measuring the cochlear microphonic potential is particularly interesting because error sources due to impaired hearing are eliminated [259]. Doubts regarding the accuracy of estimating the ICP via tympanic membrane pressure curves were raised in a baseline study [260].

A new system based on tympanic membrane temperature was developed and indicated excellent results for predicting increased ICPs and a correlation with invasive ICP monitoring (r^2^ = 0.93) [261]. 

Approaches for non-invasive ICP measurements via the tympanic membrane have been pursued for more than 35 years but remain clinically inapplicable; therefore, doubts have been cast regarding its feasibility.

#### 3.5.2. Otic Transcranial Acoustic Signals

The idea of ear-to-ear ultrasound waves is strongly related to a previously mentioned concept [128] and was introduced in 2016 [262]. Although a preliminary report [263] predicted promising results with ear pressure sensors (HS-1000, HeadSense Medical Ltd., Netanya, Israel), a prospective study demonstrated only a moderate correlation (r = 0.604) between the estimated and real ICP [264]. 

### 3.6. Short Summary of Instruments and Devices Tested in Clinical Practice

In the 1970s, invasive measurement probes were used for non-invasive purposes in infants through the fontanelle. Later, software tools (such as ICM) were developed for multimodal monitoring, which were able to estimate missing parameters, e.g., ICP curves. In the late 1980s, the flow parameters of transcranial Doppler were analyzed and correlated with the ICP. In Austria, a dielectric approach was tested in neurosurgical patients.

In 2005, the two-depth Doppler method was introduced. In the past decade, new technologies have increasingly been developed but most require further evaluation. The selected methods are listed in Table 3.

## 4. Conclusions

What levels of accuracy and reliability would be acceptable for a non-invasive method to be considered appropriate for routine clinical use? The main problem is that reliability is necessary for critical events, such as cerebral hemorrhage, seizure, or hydrocephalus. A reliability of 99% in an unremarkable intensive care stay is useless if the method would miss these critical events. Fear among intensive care physicians is one reason these procedures have not yet been incorporated into everyday clinical practice. Whether a long-term test under physiological conditions would enable a method for detecting pathological changes in a system as complex as the human body remains unclear. However, even with the established invasive test procedures, these critical events are sometimes overlooked or considered measurement errors.

MRI-ICP and two-depth Doppler (the most complex methods) show the most promising accuracy but are not always applicable. Tympanic membrane temperature, diffuse correlation spectroscopy, and natural resonance frequency are also techniques of interest, although whether the excellent correlations observed in pilot studies can be sustained remains to be seen. The retinal vein approach appears to remain an insider tip despite promising results. 

### 4.1. Limitations

The comparison of approaches is often hindered by the use of different statistical evaluations (Pearson’s correlation, coefficient of determination, sensitivity, and specificity, AUC, Bland–Altman Plots, and others). Many techniques have been evaluated only in healthy people or compared with other non-invasive techniques. Such comparisons are insufficient for clinical proposes.

Selectivity studies of novel techniques, as well as clinical applications and comparisons with the gold standard of invasive ICP measurement, are lacking. Many studies have assessed feasibility or have been performed in research institutions rather than in clinical settings.

In addition, many studies have not stated the exact inclusion and exclusion criteria for participants, thus making the studies only slightly comparable overall and introducing bias.

An exception is the studies of ONSD, which were conducted in large numbers and, in some cases, were of high quality.

### 4.2. Research Gaps

Overall, there appears to be insufficient basic research, and physically oriented approaches using other parameters (e.g., weight differences of the head, magnetism, body-position changes, or coupled with intra-abdominal pressure) are notably lacking. Most approaches have focused on physiological concepts, which may have risks (anatomical variants) or insufficient accuracy and reliability, possibly because physicians in many specialist disciplines (ENT physicians, ophthalmologists, neuroradiologists, neurosurgeons, or anesthesiologists) have a narrow research focus that sometimes loses sight of the bigger picture.

For example, several anatomical norm variants of the CSF coupling of eyes and ears exist given that different manifestations of visual impairments in astronauts have been observed after long-duration space flights [43]. In particular, in the case of illness or older patients, additional interference parameters are present (hearing impairment, cataracts, etc.). Hence, such techniques will not work for everyone.

Another point is the existence of unpredictable events (power blackouts or seizures), which may de-calibrate a method, leading to inaccurate measurements. 

Almost all researchers consider their methods to be excellent, although comparisons to the gold standard or other relevant methods are often lacking. In addition, evaluations regarding real-world applicability are lacking, such as determining the number of people the method can be used on or the frequency of non-realistic errors.

### 4.3. Future Perspectives

Even if no convincing non-invasive ICP measurement currently exists, many physical properties of the head remain to be evaluated for these pressure measurements. The best solution according to current viewpoints, will probably involve combining several methods and calibrating them at intervals (e.g., performing a lumbar puncture every 5 days). 

The continuing development of non-invasive methods with suitable feedback mechanisms is necessary to strengthen the confidence of physicians and further improve the methods.

## Figures and Tables

**Table 1:** Anatomical key structures and methods (in brackets) for measurements.

Vascular Methods	Ophthalmic Methods	Otic Methods	Brain	Whole Head
- middle cerebral artery (Doppler)	- central artery (waveform analysis, two-depth-Doppler)	- tympanic membrane (displacement, temperature)	- ventricle and subarachnoid spaces (CT and MRI)	- blood and CSF circulation (NIRS and dielectric properties)
	- retina and papilla (optical coherence tomography and ophthalmoscopy)	- cochlear microphonic potential	- CSF dynamics (MRI)	- spontaneous electrical activity of the head and brain (EEG)
	- ONSD (sonography and MRI)	- ear-to-ear ultrasound		- whole head (micromotions)
	- whole optic tract (VEP)			- fontanelle (pressure)
	- retinal veins (DVA, photo-plethysmography)			
	- pupil (pupillometry)			

**Table 2:** Overview of main studies for non-invasive intracranial pressure monitoring.

Category	No. of Studies	State of the Method (1) Pilot/Animal Study (2) Pre-/Small Clinical Test (3) Large Clinical Tests	Sensitivity for Detecting Increased ICP	Specificity for Detecting Increased ICP	Correlation with Invasive ICP Coefficient r
MR-ICP	7	2	100%	100%	r = 0.95–0.98
TCD	22	3	92%	92%	r = 0.62–0.92
ONSD	68	3	83–96%	74–96%	r = 0.53–0.82
Ocular vessels	19	2	87–94%	50–92%	r = 0.74–0.99
Tympanic membrane	10	2	81–100%	96–100%	r = 0.93
Dielectric properties	9	1–2	77–90%	76–89%	r = 0.76–0.98

**Table 3:** Selected devices and instruments.

Time	Name	Inventor	Idea
1980 to present	ICM+ (and other software tools)	Cambridge Enterprise Ltd., University of Cambridge, Cambridge, UK	Multimodal flow monitoring and analysis
1988 to present	Transcranial Doppler	Several investigators	Flow pattern analysis
1990	headband electrode	Russegger and Ennemoser, Neurosurgery, Innsbruck, Austria	Dielectric properties
2000 to present	MR-ICP	Alperin et al., University of Illinois at Chicago	CSF and blood flow calculations
2005 to present	Two-depth Doppler	Health Telematics Science Institute at the Kaunas University of Technology, Kaunas, Lithuania	Two-depth Doppler measurement of the ophthalmic artery
2005 to present	Dynamic vessel analyzer	(not primarily intended for ICP estimation) Imedos, Jena, Germany	Retinal vessel pulsations
2013 to present	Non-invasive cerebrovascular autoregulation	Health Telematics Science Institute at the Kaunas University of Technology, Kaunas, Lithuania	Ultrasonic time of flight
2017 to present	HS-1000	HeadSense Medical Ltd., Netanya, Israel	Ear-to-ear ultrasound
2019 to present	Braincare	Braincare, São Carlos, Brazil	Cranial expansion
2021 to present	Micromotions pillow	Charles University in Prague and the Military University Hospital, Prague, Czech Republic	Micromotions

## Data Availability

The data used in this review are available on request (se.mueller@klinikum-stuttgart.de).
